# The Ethics of Night Shift Allocation

**DOI:** 10.5811/westjem.68392

**Published:** 2026-11-02

**Authors:** Isha Agarwal, Frank Chessa

**Affiliations:** * MaineHealth Maine Medical Center, Department of Emergency Medicine, Portland, Maine; † MaineHealth Institute for Research, Center for Interdisciplinary and Population Health Research, Portland, Maine; ‡ Tufts University School of Medicine, Boston, Massachusetts; § MaineHealth Maine Medical Center, Division of Clinical Ethics, Portland, Maine

Dear Editor:

We read with great interest the article published in December 2024 by Croteau and colleagues, “Two-year Results of an Emergency Department Night Shift Buyout Program.”^1^ The authors describe the results of a new shift trading program whereby all faculty, regardless of academic rank, could buy out of night shifts for $500 per night shift, in exchange for day and evening shifts. Bought-out nights were used to increase the salaries of nocturnists compared to non-nocturnist attendings.

While the authors describe their program as “egalitarian,” they report that faculty who fully bought out of night shifts were more likely to be male, longer tenured, and of higher academic rank. Nocturnists were on average eight years more junior to faculty who sought full night shift buy-out, and none were associate or full professors. The significance of this disparity is magnified by the known health risks of night shift work, which include cancer, cardiovascular, and all-cause mortality.^2^ The article left us with an important ethical question: Should the burden of night shift work, a known health hazard, depend on whether an individual has the financial means to avoid it?

Ethical terminology should be used cautiously and with clear definitions—incautious use can obscure injustices found in systems with a superficial ethical credibility. The authors say that their system is “egalitarian,” but also that it succeeds in “promoting equity across our faculty groups.” Typically, an egalitarian system is one that ensures an equal distribution of benefits and burdens, while an equitable system is one in which the distribution of benefits and burdens is sensitive to the needs (or other characteristics) of individuals. Thus, egalitarian systems typically stand in contrast to equitable ones. The buy-out program advocated by Croteau and colleagues can best be described as a marketplace approach based on the ethical conception of “equality of opportunity.” That is, all faculty are offered an *equal opportunity* to buy out night shift work. The resulting distributions of the benefits and burdens of night shift work result from the choices of those within the system. Equality of opportunity systems can support equity when participants start with a level playing field. The resulting distribution can then be expected to align with an individual’s preferences to the greatest extent possible consistent with a roughly equal satisfaction of preferences among all participants in the system. The problem with the described buyout system is that participants do not start on a level playing field. For example, the $500 cost of a buyout has different consequences for a junior faculty member with a lot of debt than for a senior faculty member with a higher salary and no debt. Mechanisms such as this likely played out in the program outcomes, which resulted in more buyout of night shifts by senior, male faculty members.

A different type of ethical concern arises in discussions about what is appropriate to commodify as a good or service that is for sale. Consider the debate about whether to allow markets for organ transplantation. Proponents argue that a marketplace would incentivize organ donation and solve the current shortage of donor organs. Opponents argue a moral principle: that the human body should not be treated as an object of commerce.^3^ If we are skeptical of markets for organ transplantation, should we similarly be skeptical of buying and selling of night shift work, which carries known health risks?

Perhaps instead of thinking of night shift work as an unpleasant commodity to buy and sell, it should be understood as a civic duty, or shared professional responsibility, among emergency physicians. Such a mindset recognizes that night shift work is an unavoidable aspect of our specialty, one that is best borne collectively rather than shifted to those with the least seniority or greatest financial need. We suspect that the popularity of the buyout program described by Croteau and colleagues was not due to more equitable distribution of night shift work but rather an increase in autonomy and control over the scheduling compared to their prior rule-based system. Autonomy and control may well be the biggest ethical advantage of the buyout system. As departments seek innovative approaches to night shift scheduling and compensation, we believe they should ask not only what works, but also what values those systems reinforce.
